# Colonization Resistance of the Gut Microbiota against *Clostridium difficile*

**DOI:** 10.3390/antibiotics4030337

**Published:** 2015-08-07

**Authors:** Ana Elena Pérez-Cobas, Andrés Moya, María José Gosalbes, Amparo Latorre

**Affiliations:** 1 Joint Research Unit of Foundation for the Promotion of Health and Biomedical Research of Valencian Region (FISABIO) and the Cavanilles Institute of Biodiversity and Evolutionary Biology (ICBiBE) of the University of Valencia, Valencia 46020, Spain; E-Mails: apeco@alumni.uv.es (A.E.P.-C.); andres.moya@uv.es (A.M.); maria.jose.gosalbes@uv.es (M.J.G.); 2 CIBER in Epidemiology and Public Health (CIBERESP), Madrid 28029, Spain

**Keywords:** antibiotics, *Clostridium difficile*, gut microbiota restoration, colonization resistance

## Abstract

Antibiotics strongly disrupt the human gut microbiota, which in consequence loses its colonization resistance capacity, allowing infection by opportunistic pathogens such as *Clostridium difficile*. This bacterium is the main cause of antibiotic-associated diarrhea and a current problem in developed countries, since its incidence and severity have increased during the last years. Furthermore, the emergence of antibiotic resistance strains has reduced the efficiency of the standard treatment with antibiotics, leading to a higher rate of relapses. Here, we review recent efforts focused on the impact of antibiotics in the gut microbiome and their relationship with *C. difficile* colonization, as well as, in the identification of bacteria and mechanisms involved in the protection against *C. difficile* infection. Since a healthy gut microbiota is able to avoid pathogen colonization, restoration of the gut microbiota seems to be the most promising approach to face *C. difficile* infection, especially for recurrent cases. Therefore, it would be possible to design probiotics for patients undergoing antimicrobial therapies in order to prevent or fight the expansion of the pathogen in the gut ecosystem.

## 1. Introduction

*Clostridium difficile* is a Gram-positive, spore-forming anaerobic bacterium and is the agent that causes *C. difficile* infection (CDI) [1]. The first confirmed case of CDI was reported in 1977 and its incidence has continued rising since then, and at present it is the most frequent cause of nosocomial diarrhea worldwide, representing around 20% of cases in hospitals [2,3]. The major risk factor for CDI is antibiotic exposure, especially the use of broad-spectrum antibiotics with activity against anaerobes [4,5], but also old age (>65 years), hospitalization and an immunocompromised status can also be contributing factors [6,7]. Moreover, the use of proton-pump inhibitors, H2 antagonists and methotrexate has been associated with a higher rate of CDI, as well as the presence of some gastrointestinal problems such as inflammatory bowel disease [8,9,10].

The development of high-throughput sequencing technologies has revealed the importance of the gut microbiome for human health, including its role in avoiding the infection by opportunistic pathogens (called colonization resistance capacity). Disruption of the microbiota by antimicrobial therapies results in a higher susceptibility to infection by harmful microorganisms such as *Salmonella* spp., *Klebsiella oxytoca* or *C. difficile* [11,12,13,14].

The pathogenesis of *C. difficile* generally starts with the disruption of the normal gut microbiota equilibrium, thus reducing its colonization resistance capacity. Then, pathogen spores of endogenous or exogenous origin germinate and grow into vegetative cells. The pathogen penetrates the mucus layer and adheres to the enterocytes, colonizing the gastrointestinal tract [4]. The next phase of the pathogenesis is the production of toxins A and B encoded by the genes *tcd*A and *tcd*B. Those genes are located in the pathogenicity locus PaLoc along with the *tcd*E gene encoding a putative holin and the genes *tcd*R and *tcd*C encoding regulatory proteins [15,16]. The toxins TcdA and TcdB modify the actin cytoskeleton of epithelial cells leading to cytoskeleton disorganization, inhibition of cell division and membrane trafficking, resulting in the destruction of the intestinal epithelial cells and induction of inflammatory responses [17,18,19,20,21]. Some toxinogenic strains (around 23% of *C. difficile* isolates) present a binary toxin called CDT (encoded by *cdtA* and *cdtB* in the *Cdt* locus) that also leads to cytoskeleton disorganization [22]. This toxin is considered an additional virulence factor since it potentiates the toxicity of TcdA and TcdB, causing a more severe disease [23,24]. The most common initial treatments for CDI are oral antibiotic therapies, mainly vancomycin and metronidazole [25], although a high number of patients (between 20% and 35%) develop recurrent illnesses [26,27].

Here, we review the interplay between antibiotic treatment and *C. difficile* and the different bacteria and mechanisms operating in the gut microbiome related to the protection against *C. difficile*. Such knowledge may give us clues for the design of probiotics for patients under antimicrobial therapies, preventing the expansion of *C. difficile* and consequent infection.

## 2. Overview of the Human Gut Microbiota

The human body harbors complex microbial communities with around 10^14^ microbial symbionts, outnumbering the human cells by at least 10-fold [28]. The majority of microorganisms and the largest diversity are found in the intestinal tract, which contains on average 10^12^ cells per gram of faces and around 100-fold more genes than the human genome [28,29,30]. The collective genomes of our indigenous microbes is defined as the microbiome. The gut microbiota of adult individuals is unique and relatively stable during long periods of time (in the absence of disturbances), and the composition is influenced by several factors such as host genetics, diet, or host age [31,32,33,34,35,36].

Despite that a limited number of phyla (mainly Firmicutes, Bacteroidetes, Actinobacteria and Proteobacteria) are found in the human intestine, it harbors a high diversity of species and strains [37,38]. Some of the most prevalent and abundant genera in the human gut are *Faecalibacterium*, *Roseburia*, *Ruminococcus* (Firmicutes), *Bacteroides*, *Alistipes*, *Parabacteroides* (Bacteroidetes) or *Bifidobacterium* (Actinobacteria) [39,40]. Members of these genera are essential for host physiology. For instance, species of *Bacteroides* specialize in degrading a wide range of dietary carbohydrates undigested by the human [41,42,43,44,45]. In addition, some of the most common genera from Firmicutes, such as *Roseburia*, *Ruminococcus* or *Coprococcus,* are active producers of short-chain fatty acids (SCFAs) such as acetate, propionate and butyrate [46,47]. These molecules are absorbed by the host and provide around the 10% of calories obtained from the daily diet [48,49]. Additionally, SCFAs produced by gut bacteria also regulate the epithelial cell growth and differentiation and stimulate the immune system [50,51,52,53].

The genetic potential of the human gut microbiota has been deeply examined in recent years through “omics” approaches, such as metagenomics, metatranscriptomics or metaproteomics [39,43,54,55,56,57,58,59]. The great variation detected by these techniques regarding bacterial composition calls into question the existence of a core microbiota. Nevertheless, the great number of shared genes between individuals allows identifying a core microbiome with a high functional redundancy [57,58,60,61]. The human gut microbiome comprises a large number of genes involved in the biodegradation of dietary complex sugars and glycans that are indigestible by the host, as well as synthesis of some essential amino acids and vitamins, or detoxification of xenobiotics. These functions are important for the maintenance of the equilibrium of the gut microbial ecosystem and for host-bacterial interactions, most of them not only present, but also enriched in our gut microbiome [43,54,55,56,57,59]. As we previously discussed, a relevant function of the gut microbiota is to avoid pathogen colonization and consequent infection. In this sense, multiple levels of defense of the gut microbiota have been proposed, mainly through direct interactions between microorganisms such as competence or metabolic exclusion, and also via indirect mechanisms by stimulating the host immune system (known as immune-mediated colonization resistance) [11,62,63].

## 3. Antibiotic Effects on Diversity and Composition of the Gut Microbiota

Most of the studies regarding antibiotic effects on the gut microbiota in animals and humans have described large variations in the relative abundance of bacterial taxa during antimicrobial therapies, and a reduced diversity of the total microbiota [64,65,66,67,68,69,70,71,72]. Antibiotic therapies also have long-term effects on the community structure, with a lower abundance of specific bacterial groups, as well as an increase in the risk of antibiotic resistance genes and its transference to pathogens [66,67,68,69,72,73]. However, the gut microbiota, as a whole, is considered relatively resilient to antibiotics challenge and after short-term therapy, it returns to the pre-treatment state [74].

So far, Dethlefsen and colleagues carried out the two longest follow-up studies about antibiotic effects on human gut diversity and composition [66,67]. They studied the effects of the commonly used antibiotic oral ciprofloxacin on the fecal microbiota of different individuals by sampling their microbiota before, during and after the antimicrobial therapy. In both studies, ciprofloxacin administration correlated with a reduction of taxon richness and diversity, and the individuals showed different responses and recovery periods. Interestingly, in one of the studies, individuals were treated with a second round of ciprofloxacin and they responded differently with respect to the first course. The authors postulated that repeated disturbances have an accumulative detrimental effect on the gut microbiota, even when the community seems to recover from the initial perturbation [66]. Recent works have also addressed the effects of antibiotics on the active fraction of the gut microbiota, showing strong changes in the microbial composition, mainly in the Firmicutes phylum [71,72,75].

Although it is known that some antibiotics target specific pathogenic populations, most of the administered antibiotics in clinical practice have broad-spectrum activity and are used to treat many infections [76]. In general, the use of broad-spectrum antibiotics resulted in an increase in the relative abundance of Gram-negative bacteria as Bacteroidaceae or Enterobacteriaceae families and a decrease in the abundance of Firmicutes species [64,70,71,72,77,78]. However, the high inter-individual variability described for the human gut microbiota is reflected in the responses to antibiotics. The microbial composition of an individual strongly influences the shifts of the microbiota structure during antibiotic therapies and also the recovery process after it ends [66,67,72]. This means that the specific assembly of microorganisms of each individual interacting in diverse ways in the gut leads to a differential evolution of the microbiota under disturbance by external factors such as antibiotics.

In spite of the variability in microbial composition between individuals, the selection of resistant microorganisms after similar antibiotic treatments has led to microbial assemblies that share some features. Specifically, properties of the antibiotics as the spectrum has a large effect on the gut microbial composition. Thereby, the significant influence of the class of antibiotic on the modelling of the human gut microbiota has been recently described in individuals undergoing antibiotic therapy [72,79,80].

## 4. Antibiotic Effects on the Functional Profile of the Gut Microbiome

The antibiotic effect on the metabolic functions carried by the human gut microbiota has been poorly addressed. Mice studies showed that the microbial fermentation of carbohydrates is disrupted by broad spectrum antibiotics such as enrofloxacin, imipenem/cilastatin, gentamicin and ceftriaxone, since high concentrations of oligosaccharides (sucrose, cellobiose and raffinose), and low concentrations of monosaccharides (glucose, fucose, xylose and galactose), amino acids and SCFAs were observed in antibiotic-treated animals [81,82,83,84].

Regarding humans, in a culture-based study of metabolic activities of the gut microbiota in young adults and in elderly subjects (treated and non-treated with antibiotics), Woodmansey and colleagues showed that antibiotic-treated individuals presented a high proteolytic species diversity (Fusobacteria, Clostridia and Propionibacteria) [85]. Increased levels of protein break-down and amino acid fermentation from the metabolism of this group of bacteria resulted in the formation of toxic metabolites [86]. In addition, the presence of bacterial fermentation products that are essential to host health, specifically SCFAs (mainly butyrate, propionate, acetate), was lower in antibiotic-treated elderly. More recently, a chemostat model consisting of a defined consortium model (14 species) of the most common cultivable and saccharolytic and amino acid fermenting bacteria of the gut was used to study the effects of two broad spectrum antibiotics (metronidazole and ampicillin) on bacterial composition and metabolic activities. The two antibiotics showed great differences in their effect on the microbiota model, but for both agents, the production of SCFAs decreased while the production of some hydrolytic enzymes depended on the antibiotic-resistant bacteria [87].

Our group carried out the first follow-up study on antibiotic-associated changes of the human gut microbiota based on the integration of multiple “omics” approaches, including 16S rRNA based approaches, metagenomics, metatranscriptomics, metaproteomics and meta-metabolomics [71]. We characterized the antibiotic-associated changes of an individual undergoing beta-lactam antibiotic treatment. During the first days of treatment, the gut microbial community responded by up-regulating the expression of genes involved in avoiding the antimicrobial effects (as beta-lactamases), while during the last days of treatment the up-regulated genes were related to renewal, maintenance and repair of essential molecules (as cell wall components). The protein production was decreased during treatment, given that bacterial processes such as the glycolysis, TCA cycle, glutamate metabolism, vitamin synthesis or iron uptake were affected, leading to a lower amount of iron, sugars, branched amino acids, short organic acids or pyruvate in the gut environment, affecting the overall metabolic status of the gut ecosystem. Moreover, metabolites produced by the host and further processed by bacteria (derivatives of bile acids, cholesterol, hormones) decreased during antibiotic treatment and increased afterwards, showing how the antibiotics can alter the interplay between the liver/pancreas and colonic bacteria, possibly affecting the entero-hepatic recirculation and systemic lipid metabolism of the host [71].

On the other hand, a general mechanism of antibiotic response of the gut microbiome is related to sugar transport and metabolism, since a higher presence of genes from the phosphotransferase system (PTS) has been described in the metagenomes of a group of individuals undergoing broad-spectrum antibiotic therapies [72]. The PTS is the main sugar translocation system for bacteria and it participates in regulatory processes including stress responses [88]. In a study by Hernández and co-workers, it was found that antibiotic-treated and obese individuals showed higher and less balanced sugar anabolic capability with respect to healthy and lean subjects [89]. The beneficial effect of this function may be due to the role of sugar transporters in counteracting osmotic stress, as well as to their participation in the energy metabolism, giving the microorganisms an advantage for survival and colonization in the gut under unstable conditions.

## 5. CDI and the Gut Microbiota

The main risk for developing CDI is antibiotic use in hospitals, mainly clindamycin, cephalosporins, penicillins and more recently fluoroquinolones [90,91]. Antibiotic exposures may have long-lasting effects on the community structure of the microbiota, affecting the biomass, composition and functions of the gut microbiota, including the colonization resistance capacity [71].

The development of animal models to study CDI has led to an advance in understanding the relationship between the microbiota and the development of infection. In mice, a variety of antibiotics has been associated with the loss of resistance to CDI [65,92,93,94]. A study of CDI of antibiotic treated mice described a high abundance of Proteobacteria (mainly Enterobacteriaceae) and a low abundance of normal members of the microbiota such as Lachnospiraceae (Firmicutes) in ill animals compared with healthy controls [93]. The same group investigated, also in mice, the role of strains of both groups (Enterobacteriaceae and Lachnospiraceae) in colonization resistance against CDI [95]. They found that only the Lachnospiraceae strain was able to partially restore the colonization resistance because Lachnospiraceae-inoculated mice presented decreased *C. difficile* colonization, lower intestinal cytotoxin levels and less severe clinical signs. A different study showed that six phylogenetically diverse species of gut normal bacteria administered together were able to restore colonization resistance and prevent the chronic carriage of *C. difficile* in mice [96].

Studies of the gut microbiome on patients with CDI have provided data that correlate with some findings from animal studies. Specifically, changes in the diversity and a significant decrease in the relative abundance of some beneficial microbial groups, (*i.e.*, some Clostridiales members, mainly Lachnospiraceae and Ruminococcaceae) have been shown [97,98,99,100,101]. Chang and colleagues profiled the gut microbiota of patients with antibiotic-associated diarrhea due to *C. difficile* in both initial and recurrent episodes using 16S rRNA-encoding gene clone libraries. They found a highly variable bacterial composition and very low diversity in the recurrent patients. Patients with initial infections showed an intermediate diversity pattern between recurrent CDI patients and controls [98]. In a different study, the microbiota associated to CDI in elderly was examined by pyrosequencing of the 16S rRNA gene amplicons, and a reduced microbial diversity when comparing infected with non-infected patients was described [102]. The infected patients showed a higher abundance of Lactobacillaceae and Enterococcaceae and a lower abundance of *Bacteroides*, *Prevotella* and Bifidobacteria. A lower diversity prior to episodes of CDI was observed in a similar comparative study of patients undergoing antibiotic therapy. Bacteria from Bacteroidaceae and Clostridiales were depleted in patients compared with controls, whereas patients were enriched with the Enterococcaceae family [101]. The higher abundance of Enterococcaceae members could be explained by their opportunistic nature that, similarly to *C. difficile*, could take advantage of a low bacterial diversity to overgrow. A recent research compared the structure of the microbiota (based on 16S rRNA gene pyrosequencing) of three groups of individuals: CDI subjects with diarrhea, *C. difficile*-negative nosocomial diarrhea (CDN) and healthy controls. Significant alterations in the microbiota were associated to CDI and CDN compared with controls. Bacteria producing butyrate and other SCFAs mainly from Ruminococcaceae and Lachnospiraceae families were depleted in CDI and CDN, suggesting a main role of these bacteria in colonization resistance against the pathogen and in the maintenance of the ecosystem equilibrium [97]. Moreover, supporting the results of the discussed studies, a recent follow-up study of infected and non-infected patients undergoing broad-spectrum antibiotic therapies found that CDI samples were enriched in Enterococcaceae and Lactobacillaceae families, while Ruminococcaceae and Lachnospiraceae were under-represented [100].

## 6. CDI Current Treatments and Promising Approaches

The most effective initial treatment for CDI are antibiotic therapies, mainly metronidazole, followed by vancomycin commonly administered orally or intravenously [25,103,104]. However, a high number of patients (between 20% and 35%) develop recurrent infection several days to weeks after the initial administration of the antibiotic therapy [26,27]. A pulsed antibiotic (vancomycin/metronidazole) regimen could be used to manage a second or third recurrence. Taur and Pamer indicated that using antibiotics to cure a condition caused by antibiotics is a conceptually incorrect strategy and that it could be the reason for the high recurrence rates for CDI [105]. In fact, the common treatments for relapse cases consist in prolonged or pulsed antibiotic courses with low success [106].

An alternative method for the maintenance and restoration of the gut microbiota after antibiotic therapies involves the use of prebiotics and probiotics. Probiotics as bifidobacteria and lactobacilli seem to counteract the antibiotic-associated diarrhea and relapsing CDI, but some studies on probiotics to treat CDI have shown conflicting results [107,108]. At present, the use of probiotics is not recommended in the clinical community for the prevention of CDI [103,104]. Several other alternative therapies have been proposed such as the use of a novel neutralizing monoclonal antibody against *C. difficile* toxins, the anion-binding resins that neutralize clostridial toxins, vaccination against the pathogen and its toxins or immunoglobulin based therapies, but most of these treatments are in an early stage of development [109,110,111,112,113].

**Figure 1:**
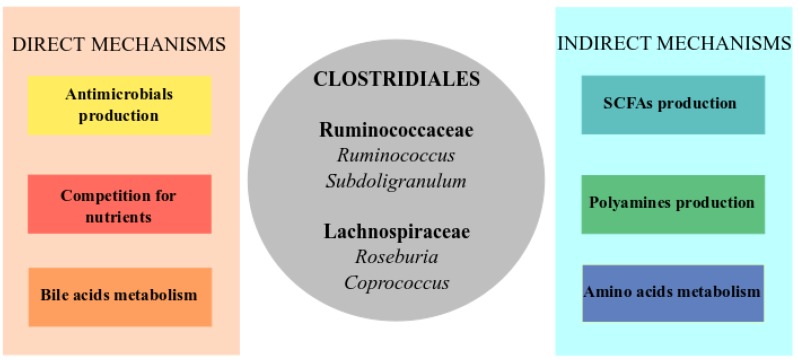
Core of microorganisms and metabolic pathways involved in colonization resistance against *C. difficile* infection (CDI). The direct mechanisms (**left** panel) include direct inhibition of the pathogen through the production of antimicrobial compounds as bacteriocins, the competition for nutrients such as sugars or metabolism of bile acids that inhibits *C. difficile* proliferation. The indirect mechanisms (**right** panel) are related to stimulation of the immune system and restoration of the intestinal equilibrium through the amino acid metabolism and production of short-chain fatty acids (SCFAs) and polyamines. In the center of the figure (grey circle), a list of genera, mainly from Clostridiales from which some commensal members have been found as protectives against CDI in most of the studies, and which could be involved in some of the represented functions.

Since a healthy gut microbiota is able to control the pathogen infection, restoration of the gut microbiota seems to be the most promising approach to face CDI, especially for recurrent cases. In this sense, a therapy known as fecal microbiota transplantation (FMT) that consists in transferring feces from a healthy donor to a patient is being performed at multiple centers around the world for treatment of recurrent CDI. FMT has been very successful, restoring the intestinal microbial diversity in up to 95% of patients [96,105,114,115,116,117,118,119]. However, the microbial ecosystem of feces is complex, and many biological processes which occur there remain unknown. This fact introduces some concerns about fecal microbial transplantation, such as a possible introduction of pathogens, or alterations of microbiota-host interactions that could trigger some microbiota-related diseases, although no evidence of these complications has been found [105,120,121,122]. In fact, a study of fecal microbiota transplantation for CDI in immunocompromised patients showed a high rate of cure and no related infectious complications, suggesting that this approach seems to be safe at least for this risk group [123]. In this regard, an alternative may be to identify the consortia of bacteria involved in pathogen protection, so that they could be administered to patients in an alternative way such as probiotics [105,122]. Thus, some recent studies focusing on the identification of bacteria involved in resistance colonization against this pathogen in both mice and humans are very promising [95,96,97,100,115,124]. Interestingly, regardless of the methods and models used, some of the bacteria under-represented in CDI samples (possibly protectors) are common to many studies, suggesting that we are close to obtaining an optimal set of bacteria that can prevent the pathogen infection (represented in Figure 1). Members of the Clostridiales, principally species from the families Ruminococcaceae and Lachnospiraceae, have been the common factor among these studies [93,95,97,100,101]. For instance, a recent research showed that 33 strains of a healthy donor were able to cure two patients with recurrent CDI, 11 strains being from the Lachnospiraceae family [115]. A mouse study showed that a strain of Lachnospiraceae was able to restore partially the gut microbiota after CDI, but the total restoration was only possible when the fecal microbial community of a healthy donor was transferred to the infected animals [95]. Some genera of these families, such as *Ruminococcus*, *Subdoligranulum* (Ruminococcaceae) and *Roseburia*, *Coprococcus* (Lachnospiraceae), could be involved in colonization resistance since they have been also depleted in CDI samples from other human studies [97,100,101]. Interestingly, unclassified OTUs belonging to these families have also appeared as possible participants of the colonization resistance response, indicating that species with a possible key role in protection could remain undescribed [95,97,100]. Recently, a clinical study showed that colonization of patients recovered from CDI with a nontoxigenic *C. difficile* strain significantly reduced CDI recurrence [125]. Moreover, since a diverse microbiota is better at fighting CDI, it has been proposed to also test bacteria from other phyla such as *Alistipes* (Bacteroidetes), *Escherichia* (Proteobacteria) or Coriobacteriaceae (Actinobacteria as protectors against CDI [100].

Future studies should focus on testing the effectiveness of different strains of these taxa (and combinations of them) in preventing CDI. This will allow designing specific probiotics for CDI individuals.

## 7. Possible Role of the Bacteria Involved in Colonization Resistance against *C. difficile*

In this section, we discuss the different mechanisms through which the protective members of the gut microbiota could avoid CDI (summarized in Figure 1).

Most of the bacteria candidates as protectors have been described as producers of SCFAs (mainly from Lachnospiraceae and Ruminococcaceae families), which are the main source of energy of the gut epithelium and have anti-inflammatory effects [62]. In addition, SCFAs stimulate defense barriers by increasing antimicrobial peptide levels and mucin production, as well as participate in the generation of a mucosal regulatory T cell subset as part of immune response [53,125]. Thus, a proposed mechanism of colonization resistance for this group of bacteria is through the production of SCFAs and the positive stimulation of the gut epithelial and immune system functions [62]. In this sense, a study that compared three groups of individuals—healthy, *C. difficile* negative with nosocomial diarrhea and *C. difficile* positive with nosocomial diarrhea—found a lower microbial diversity and abundance of SCFAs producers in the individuals with diarrhea, regardless of the infection status [97]. Hence, the depletion of these organisms leads to a dysfunction of the intestine and diarrhea, reinforcing the role of these microbial products in the correct functioning of the gut. In contrast, some studies found that SCFAs administration to infected mice did not reduce the CDI [95,126]. Therefore, it is still unknown if the SCFAs play a leading or secondary role in avoiding the CDI, but it is clear that they contribute positively to preventing and fighting pathogen infection through maintaining the gut epithelial equilibrium and stimulating human defenses.

A different mechanism by which the microbiota could participate in colonization resistance to CDI is through its role in bile acid transformation. Some *in vitro* studies have shown that primary bile acids produced by the liver and secreted in the small intestine stimulate the germination of *C. difficile* spores [127,128]. The recent identification of a bile acid receptor of *C. difficile* required for germination and colonization supports the role of bile metabolism in CDI [129]. Moreover, a fraction of the gut microbiota is involved in the transformation of primary bile acids to secondary bile acids [127,130,131]. Specifically, the action of the microbial enzyme 7-dehydroxylase allows transforming the cholate (primary bile acid) into deoxycholate (secondary bile acid). The secondary bile acids, such as deoxycholate, strongly inhibit the vegetative growth of the pathogen and reduce the *C. difficile*’s ability to colonize the gut [91,132,133,134]. Antibiotics alter the bile acid equilibrium leading to higher levels of primary bile acids (cholate) that facilitate *C. difficile* germination and overgrowth [71,81,134]. Additionally, Theriot and colleagues found in a mice model that the gut metabolome of animals susceptible to infection was characterized by a relative increase in primary bile acids and a reduced level of secondary ones [135]. In this regard, Britton and Young (2012) suggested that bacteria harbouring the enzyme responsible of primary bile acid transformation (7-dehydroxylase) may be potential probiotics against *C. difficile* colonization and infection [91]. It is worth mentioning that the dehydrolase activity in the gut has been described mainly for members of Clostridia as *Clostridium* and *Eubacterium* genera [136,137]. Since many of the identified candidate bacteria to protect against CDI have belonged to Clostridia, it is possible that their role in colonization resistance could be related to the metabolism of bile acids. However, it is still unknown which gut microbial members contribute the most to this function *in vivo*. Further studies should focus on testing the activity of different gut bacterial species regarding bile acid transformation.

Since the gut microbiota forms a complex metabolic network where the product of the metabolism of a specific microorganism is the substrate for another, competition for niches and nutrients has been proposed as a resistance mechanism against the establishment of pathogens [62,91]. For instance, studies with pathogenic *E. coli* strains showed that these bacteria compete with commensal *E. coli* strains for nutrients (*i.e.*, sugars) and that commensal members are able to prevent the growth of the pathogenic strains in the mouse intestine [138,139]. In the case of *C. difficile*, some studies pointed to the competition for available resources, mainly carbohydrates, as a colonization resistance mechanism [140,141,142,143].

In a preliminary study based on continuous flow-cultures, it was found that components of the mucin (sialic acids, N-acetylglucosamine, N-acetylneuraminic acid) are required to promote the expansion of intestinal bacteria able to suppress *Clostridium* [140]. Recently, a transcriptomic study showed that *C. difficile* is able to use sialic acids as a carbon source to reach high densities and colonize the mice intestine [142]. The authors postulated that when the normal gut microbiota is reduced by antibiotics, sialic acids are liberated with sufficient bacteria with sialidases, but as there are less sialic acid consumers (competitors), *C. difficile* has a greater chance of achieving colonization. In a recent work by our group, we found an over-representation of sugar transporters in the metagenomes of CDI samples compared with those non-infected, indicating that an alteration of the carbohydrates’ metabolism of a disrupted microbiota could favour *C. difficile* colonization [100]. Taking into account that the candidate bacteria that we proposed are phylogenetically related to *Clostridium*, they might share niches and compete for similar resources. This idea is supported by a study where a non-toxigenic *C. difficile* strain was able to prevent the establishment of a toxigenic *C. difficile* since it was more successful at competing for limited resources [141,143].

A direct mechanism of colonization resistance by the gut microbiota against CDI could be the production of antimicrobials that inhibit the pathogen growth. Different antimicrobials produced by the gut microbiota are capable of killing pathogens including *C. difficile* [62,91]. An intestinal strain of *Bacillus thuringensis* produces the bacteriocin Thuricin CD that has a narrow activity against *C. difficile* [144]. Although the activity of this bacteriocin was tested *in vitro*, this capacity could represent a possible defense of specific members of the microbiota against specific pathogens.

The cross-talk between human immune system and gut microbiota could be an indirect colonization resistance mechanism against CDI. The innate and adaptive immune responses of the host are important to reduce the acute inflammation associated to CDI, as well as the recurrence of the disease [145,146,147]. Moreover, these immune-associated responses are at least partly dependent on beneficial bacteria that stimulate the immune system, which in turn targets the pathogen [11]. However, antibiotic treatments reduce microbial diversity and by affecting the microbiota-immune system interactions they suppress the innate immune system, creating an ideal environment in the host for opportunistic pathogens such as *C. difficile* [148]. The protective group of commensal members of Clostridia exerts a strong influence on development of the host immune system [73,149,150]. It has been described that some beneficial Clostridia species promote the development of T-cell receptor, intraepithelial lymphocytes (IEL) and immunoglobulin A (IgA-). The stimulation of the immune system by this group seems to work through a gradient of SCFAs and secondary bile acids that are sensed by the epithelial cells, triggering the initiation of immunological signaling [151,152]. In relation to the immune system stimulation and the gut epithelial homeostasis, the products of some metabolic pathways related to amino acids’ metabolism (tryptophan or polyamines biosynthesis) have been proposed to be protective against CDI [100]. Specifically, an increase in polyamine levels is associated with a major growth of intestinal mucosal cells and microorganisms, possibly contributing to restoration of the gut equilibrium during stress conditions, and they also have important anti-inflammatory effects [153,154]. The possible protective role of these metabolites against CDI is supported by a recent meta-metabolomic study in which CDI patients showed a deficiency in the production of polyamines with respect to healthy controls [155].

## 8. Conclusions

Antibiotic treatment depletes members of the gut microbiota that through colonization resistance mechanisms are able of preventing CDI. The gut environment under antibiotic stress contributes to *C. difficile* germination and growth, with a low abundance of SCFAs, high abundance of primary bile acids, high carbohydrate availability, a suppressed immune system and absence of competitors. On the other hand, the metabolism of commensal members of Clostridiales in the gut contributes to create an adverse environment for *C. difficile* germination and colonization, since they have a strong influence in physiologic, metabolic and immunological processes in the gut. The FMT has shown promising results in curing CDI, but the dynamics of the gut microbial ecosystem is complex and this treatment could bring some unexpected complications for the host health. Future studies should reveal the colonization resistance mechanism(s) operating in the gut ecosystem, in which microbial members are mainly involved. It will be possible to focus on the capacity of key species of the gut in fighting CDI with the possibility of administering then to the affected individuals. Thereby, hopefully, it will be possible to design specific diets, prebiotics or probiotics for patients undergoing antimicrobial therapies in order to prevent and fight CDI, especially in recurrent cases.

## References

[1] Bartlett J.G. (1994). *Clostridium difficile*: History of its role as an enteric pathogen and the current state of knowledge about the organism. Clin. Infect. Dis..

[2] Bartlett J.G., Gerding D.N. (2008). Clinical recognition and diagnosis of *Clostridium difficile* infection. Clin. Infect. Dis..

[3] Kuijper E.J., Dissel J.T., Wilcox M.H. (2007). *Clostridium difficile*: Changing epidemiology and new treatment options. Curr. Opin. Infect. Dis..

[4] Denève C., Janoir C., Poilane I., Fantinato C., Collignon A. (2009). New trends in *Clostridium difficile* virulence and pathogenesis. Int. J. Antimicrob. Agents.

[5] McFarland L.V. (2008). Antibiotic-associated diarrhea: Epidemiology, trends and treatment. Future Microbiol..

[6] Keller J.M., Surawicz C.M. (2014). *Clostridium difficile* infection in the elderly. Clin. Geriatr. Med..

[7] Simor A.E., Bradley S.F., Strausbaugh L.J., Crossley K., Nicolle L.E. (2002). SHEA Long-Term-Care Committee. *Clostridium difficile* in long-term-care facilities for the elderly. Infect. Control Hosp. Epidemiol..

[8] Dial S., Delaney J.A.C., Barkun A.N., Suissa S. (2005). Use of gastric acid-suppressive agents and the risk of community-acquired *Clostridium difficile*-associated disease. JAMA.

[9] Dial S., Delaney J.A., Schneider V., Suissa S. (2006). Proton pump inhibitor use and risk of community-acquired *Clostridium difficile*-associated disease defined by prescription for oral vancomycin therapy. CMAJ.

[10] Nguyen G.C., Kaplan G.G., Harris M.L., Brant S.R. (2008). A national survey of the prevalence and impact of *Clostridium difficile* infection among hospitalized inflammatory bowel disease patients. Am. J. Gastroenterol..

[11] Buffie C.G., Pamer E.G. (2013). Microbiota-mediated colonization resistance against intestinal pathogens. Nat. Rev. Immunol..

[12] Donskey C.J. (2006). Antibiotic regimens and intestinal colonization with antibiotic-resistant gram-negative bacilli. Clin. Infect. Dis..

[13] Taur Y., Xavier J.B., Lipuma L., Ubeda C., Goldberg J., Gobourne A., Lee Y.J., Dubin K.A., Socci N.D., Viale A. (2012). Intestinal domination and the risk of bacteremia in patients undergoing allogeneic hematopoietic stem cell transplantation. Clin. Infect. Dis..

[14] Willing B.P., Russell S.L., Finlay B.B. (2011). Shifting the balance: Antibiotic effects on host-microbiota mutualism. Nat. Rev. Microbiol..

[15] Dupuy B., Govind R., Antunes A., Matamouros S. (2008). *Clostridium difficile* toxin synthesis is negatively regulated by TcdC. J. Med. Microbiol..

[16] Tan K.S., Wee B.Y., Song K.P. (2001). Evidence for holin function of *tcdE* gene in the pathogenicity of *Clostridium difficile*. J. Med. Microbiol..

[17] Jaffe A.B., Hall A. (2005). Rho GTPases: Biochemistry and biology. Annu. Rev. Cell Dev. Biol..

[18] Just I., Selzer J., Eichel-Streiber C., Aktories K. (1995). The low molecular mass GTP-binding protein Rho is affected by toxin A from *Clostridium difficile*. J. Clin. Investig..

[19] Just I., Selzer J., Wilm M., Eichel-Streiber C., Mann M., Aktories K. (1995). Glucosylation of Rho proteins by *Clostridium difficile* toxin B. Nature.

[20] Ridley A.J. (2001). Rho family proteins: Coordinating cell responses. Trends Cell Biol..

[21] Voth D., Ballard J. (2005). *Clostridium difficile* toxins: Mechanism of action and role in disease. Clin. Microbiol. Rev..

[22] Gerding D.N., Johnson S., Rupnik M., Aktories K. (2014). *Clostridium difficile* binary toxin CDT: Mechanism, epidemiology, and potential clinical importance. Gut Microbes.

[23] Barbut F., Decré D., Lalande V., Burghoffer B., Noussair L., Gigandon A., Espinasse F., Raskine L., Robert J., Mangeol A. (2005). Clinical features of *Clostridium difficile*-associated diarrhoea due to binary toxin (actin-specific ADP-ribosyltransferase)-producing strains. J. Med. Microbiol..

[24] Gerding D.N., Johnson S., Rupnik M., Aktories K. (2014). *Clostridium difficile* binary toxin CDT: mechanism, epidemiology, and potential clinical importance. Gut Microbes.

[25] Miller M.A. (2007). Clinical management of *Clostridium difficile*-associated disease. Clin. Infect. Dis..

[26] McFarland L.V., Elmer G.W., Surawicz C.M. (2002). Breaking the cycle: Treatment strategies for 163 cases of recurrent *Clostridium difficile* disease. Am. J. Gastroenterol..

[27] Wilcox M.H. (1998). *Clostridium difficile* infection: Appendix. J. Antimicrob. Chemother..

[28] Savage D.C. (1977). Microbial ecology of the gastrointestinal tract. Annu. Rev. Microbiol..

[29] Hooper L., Gordon J.I. (2001). Commensal host-bacterial relationships in the gut. Science.

[30] Yang X., Xie L., Li Y., Wei C. (2009). More than 9,000,000 unique genes in human gut bacterial community: Estimating gene numbers inside a human body. PLoS ONE.

[31] Claesson M.J., Cusack S., O’Sullivan O., Greene-Diniz R., Weerd H., Flannery E., Marchesi J.R., Falush D., Dinan T., Fitzgerald G. (2011). Composition, variability, and temporal stability of the intestinal microbiota of the elderly. Proc. Natl. Acad. Sci. USA.

[32] David L.A., Maurice C.F., Carmody R.N., Gootenberg D.B., Button J.E., Wolfe B.E., Ling A.V., Devlin A.S., Varma Y., Fischbach M.A. (2013). Diet rapidly and reproducibly alters the human gut microbiome. Nature.

[33] Dethlefsen L., Eckburg P.B., Bik E.M., Relman D.A. (2006). Assembly of the human intestinal microbiota. Trends Ecol. Evol..

[34] Durbán A., Abellán J.J., Jiménez-Hernández N., Latorre A., Moya A. (2012). Daily follow-up of bacterial communities in the human gut reveals stable composition and host-specific patterns of interaction. FEMS Microbiol. Ecol..

[35] Vallès Y., Artacho A., Pascual-García A., Ferrús M.L., Gosalbes M.J., Abellán J.J., Francino M.P. (2014). Microbial succession in the gut: Directional trends of taxonomic and functional change in a birth cohort of spanish infants. PLoS Genet..

[36] Zoetendal E.G., Akkermans A.D., Vos W.M. (1998). Temperature gradient gel electrophoresis analysis of 16S rRNA from human fecal samples reveals stable and host-specific communities of active bacteria. Appl. Environ. Microbiol..

[37] Eckburg P.B., Bik E.M., Bernstein C.N., Purdom E., Dethlefsen L., Sargent M., Gill S.R., Nelson K.E., Relman D.A. (2005). Diversity of the human intestinal microbial flora. Science.

[38] Ley R.E., Lozupone C., Hamady M., Knight R., Gordon J.I. (2008). Worlds within worlds: Evolution of the vertebrate gut microbiota. Nat. Rev. Microbiol..

[39] Arumugam M., Raes J., Pelletier E., Paslier D., Yamada T., Mende D.R., Fernandes G.R., Tap J., Bruls T., Batto J.M. (2011). Enterotypes of the human gut microbiome. Nature.

[40] Tap J., Mondot S., Levenez F., Pelletier E., Caron C., Furet J.P., Ugarte E., Muñoz-Tamayo R., Paslier D.L., Nalin R. (2009). Towards the human intestinal microbiota phylogenetic core. Environ. Microbiol..

[41] Bäckhed F., Ley R.E., Sonnenburg J.L., Peterson D.A., Gordon J.I. (2005). Host-bacterial mutualism in the human intestine. Science.

[42] Cho K.H., Salyers A.A. (2001). Biochemical analysis of interactions between outer membrane proteins that contribute to starch utilization by *Bacteroides thetaiotaomicron*. J. Bacteriol..

[43] Kurokawa K., Itoh T., Kuwahara T., Oshima K., Toh H., Toyoda A., Takami H., Morita H., Sharma V.K., Srivastava T.P. (2007). Comparative metagenomics revealed commonly enriched gene sets in human gut microbiomes. DNA Res..

[44] Xu J., Bjursell M.K., Himrod J., Deng S., Carmichael L.K., Chiang H.C., Hooper L.V., Gordon J.I. (2003). A genomic view of the human-*Bacteroides thetaiotaomicron* symbiosis. Science.

[45] Zocco M.A., Ainora M.E., Gasbarrini G., Gasbarrini A. (2007). *Bacteroides thetaiotaomicron* in the gut: Molecular aspects of their interaction. Dig. Liver Dis..

[46] Barcenilla A., Pryde S.E., Martin J.C., Duncan S.H., Stewart C.S., Henderson C., Flint H.J. (2000). Phylogenetic relationships of butyrate-producing bacteria from the human gut. Appl. Environ. Microbiol..

[47] Pryde S.E., Duncan S.H., Hold G.L., Stewart C.S., Flint H.J. (2002). The microbiology of butyrate formation in the human colon. FEMS Microbiol. Lett..

[48] Leser T.D., Molbak L. (2009). Better living through microbial action: The benefits of the mammalian gastrointestinal microbiota on the host. Environ. Microbiol..

[49] Turroni F., Ribbera A., Foroni E., Sinderen D., Ventura M. (2008). Human gut microbiota and bifidobacteria: From composition to functionality. Antonie Leeuwenhoek.

[50] Fung K.Y.C., Cosgrove L., Lockett T., Head R., Topping D.L. (2012). A review of the potential mechanisms for the lowering of colorectal oncogenesis by butyrate. Br. J. Nutr..

[51] Matsuki T., Pédron T., Regnault B., Mulet C., Hara T., Sansonetti P.J. (2013). Epithelial cell proliferation arrest induced by lactate and acetate from *Lactobacillus casei* and *Bifidobacterium breve*. PLoS ONE.

[52] O’Keefe S.J.D. (2008). Nutrition and colonic health: The critical role of the microbiota. Curr. Opin. Gastroenterol..

[53] Wong J.M.W., Souza R., Kendall C.W.C., Emam A., Jenkins D.J.A. (2006). Colonic health: Fermentation and short chain fatty acids. J. Clin. Gastroenterol..

[54] Gill S.R., Pop M., Deboy R.T., Eckburg P.B., Turnbaugh P.J., Samuel B.S., Gordon J.I., Relman D.A., Fraser-Liggett C.M., Nelson K.E. (2006). Metagenomic analysis of the human distal gut microbiome. Science.

[55] Gosalbes M.J., Durbán A., Pignatelli M., Abellan J.J., Jiménez-Hernández N., Pérez-Cobas A.E., Latorre A., Moya A. (2011). Metatranscriptomic approach to analyze the functional human gut microbiota. PLoS ONE.

[56] Kolmeder C.A., de Been M., Nikkilä J., Ritamo I., Mättö J., Valmu L., Salojärvi J., Palva A., Salonen A., de Vos W.M. (2012). Comparative metaproteomics and diversity analysis of human intestinal microbiota testifies for its temporal stability and expression of core functions. PLoS ONE.

[57] Qin J., Li R., Raes J., Arumugam M., Burgdorf K.S., Nielsen T., Pons N., Levenez F., Yamada T., Mende D.R. (2010). A human gut microbial gene catalogue established by metagenomics sequencing. Nature.

[58] Turnbaugh P.J., Quince C., Faith J.J., McHardy A.C., Yatsunenko T., Niazi F., Affourtit J., Egholm M., Henrissat B., Knight R. (2009). Organismal, genetic, and transcriptional variation in the deeply sequenced gut microbiomes of identical twins. Proc. Natl. Acad. Sci. USA.

[59] Verberkmoes N.C., Russell A.L., Shah M., Godzik A., Rosenquist M., Halfvarson J., Lefsrud M.G., Apajalahti J., Tysk C., Hettich R.L. (2009). Shotgun metaproteomics of the human distal gut microbiota. ISME J..

[60] Lozupone C., Stombaugh J., Gordon J., Jansson J., Knight R. (2012). Diversity, stability and resilience of the human gut microbiota. Nature.

[61] Human Microbiome Project Consortium (2012). Structure, function and diversity of the healthy human microbiome. Nature.

[62] Lawley T.D., Walker A.W. (2013). Intestinal colonization resistance. Immunology.

[63] Stecher B., Hardt W.D. (2011). Mechanisms controlling pathogen colonization of the gut. Curr. Opin. Microbiol..

[64] Antonopoulos D.A., Huse S.M., Morrison H.G., Schmidt T.M., Sogin M.L., Young V.B. (2009). Reproducible community dynamics of the gastrointestinal microbiota following antibiotic perturbation. Infect. Immun..

[65] Buffie C.G., Jarchum I., Equinda M., Lipuma L., Gobourne A., Viale A., Ubeda C., Xavier J., Pamer E.G. (2012). Profound alterations of intestinal microbiota following a single dose of clindamycin results in sustained susceptibility to *Clostridium difficile*-induced colitis. Infect. Immun..

[66] Dethlefsen L., Relman D.A. (2011). Incomplete recovery and individualized responses of the human distal gut microbiota to repeated antibiotic perturbation. Proc. Natl. Acad. Sci. USA.

[67] Dethlefsen L., Huse S., Sogin M.L., Relman D.A. (2008). The pervasive effects of an antibiotic on the human gut microbiota, as revealed by deep 16S rRNA sequencing. PLoS Biol..

[68] Jakobsson H.E., Jernberg C., Andersson A.F., Sjölund-Karlsson M., Jansson J.K., Engstrand L. (2010). Short-term antibiotic treatment has differing long-term impacts on the human throat and gut microbiome. PLoS ONE.

[69] Jernberg C., Löfmark S., Edlund C., Jansson J.K. (2007). Long-term ecological impacts of antibiotic administration on the human intestinal microbiota. ISME J..

[70] Panda S., El khader I., Casellas F., López Vivancos J., García Cors M., Santiago A., Cuenca S., Guarner F., Manichanh C. (2014). Short-term effect of antibiotics on human gut microbiota. PLoS ONE.

[71] Pérez-Cobas A.E., Gosalbes M.J., Friedrichs A., Knecht H., Artacho A., Eismann K., Otto W., Rojo D., Bargiela R., von Bergen M. (2013). Gut microbiota disturbance during antibiotic therapy: A multi-omic approach. Gut.

[72] Pérez-Cobas A.E., Artacho A., Knecht H., Ferrús M.L., Friedrichs A., Ott S.J., Moya A., Latorre A., Gosalbes M.J. (2013). Differential Effects of Antibiotic Therapy on the Structure and Function of Human Gut Microbiota. PLoS ONE.

[73] Francino M.P., Moya A. (2013). Effects of antibiotic use on the microbiota of the gut and associated alterations of immunity and metabolism. EMJ Gastroenterol..

[74] Cochetière M.F., Durand T., Lepage P., Bourreille A., Galmiche J.P., Dore J. (2005). Resilience of the dominant human fecal microbiota upon short-course antibiotic challenge. J. Clin. Microbiol..

[75] Maurice C.F., Haiser H.J., Turnbaugh P.J. (2013). Xenobiotics shape the physiology and gene expression of the active human gut microbiome. Cell.

[76] Nathan C. (2004). Antibiotics at the crossroads. Nature.

[77] Pham T.A.N., Lawley T.D. (2014). Emerging insights on intestinal dysbiosis during bacterial infections. Curr. Opin. Microbiol..

[78] Theriot C.M., Young V.B. (2014). Microbial and metabolic interactions between the gastrointestinal tract and *Clostridium difficile* infection. Gut Microbes.

[79] Looft T., Johnson T.A., Allen H.K., Bayles D.O., Alt D.P., Stedtfeld R.D., Sul W.J., Stedtfeld T.M., Chai B., Cole J.R. (2012). In-feed antibiotic effects on the swine intestinal microbiome. Proc. Natl. Acad. Sci. USA.

[80] O’Sullivan O., Coakley M., Lakshminarayanan B., Conde S., Claesson M.J., Cusack S., Fitzgerald A.P., O’Toole P.W., Stanton C., Ross R.P. (2013). Alterations in intestinal microbiota of elderly Irish subjects post-antibiotic therapy. J. Antimicrob. Chemother..

[81] Antunes L.C.M., Han J., Ferreira R.B.R., Lolić P., Borchers C.H., Finlay B.B. (2011). Effect of antibiotic treatment on the intestinal metabolome. Antimicrob. Agents Chemother..

[82] Romick-Rosendale L.E., Goodpaster A.M., Hanwright P.J., Patel N.B., Wheeler E.T., Chona D.L., Kennedy M.A. (2009). NMR-based metabonomics analysis of mouse urine and fecal extracts following oral treatment with the broad-spectrum antibiotic enrofloxacin (Baytril). Magn. Reson. Chem..

[83] Zhao Y., Wu J., Li J.V., Zhou N.Y., Tang H., Wang Y. (2013). Gut microbiota composition modifies fecal metabolic profiles in mice. J. Proteome Res..

[84] Zheng X., Xie G., Zhao A., Zhao L., Yao C., Chiu N.H., Zhou Z., Bao Y., Jia W., Nicholson J.K. (2011). The footprints of gut microbial-mammalian co-metabolism. J. Proteome Res..

[85] Woodmansey E.J., McMurdo M.E.T., Macfarlane G.T., Macfarlane S. (2004). Comparison of compositions and metabolic activities of fecal microbiotas in young adults and in antibiotic-treated and non-antibiotic-treated elderly subjects. Appl. Environ. Microbiol..

[86] Macfarlane G.T., Cummings J.H., Macfarlane S., Gibson G.R. (1989). Influence of retention time on degradation of pancreatic enzymes by human colonic bacteria grown in a 3-stage continuous culture system. J. Appl. Bacteriol..

[87] Newton D.F., Macfarlane S., Macfarlane G.T. (2013). Effects of antibiotics on bacterial species composition and metabolic activities in chemostats containing defined populations of human gut microorganisms. Antimicrob. Agents Chemother..

[88] Deutscher J., Francke C., Postma P.W. (2006). How phosphotransferase system-related protein phosphorylation regulates carbohydrate metabolism in bacteria. Microbiol. Mol. Biol. Rev..

[89] Hernández E., Bargiela R., Diez M.S., Friedrichs A., Pérez-Cobas A.E., Gosalbes M.J., Knecht H., Martínez-Martínez M., Seifert J., von Bergen M. (2013). Functional consequences of microbial shifts in the human gastrointestinal tract linked to antibiotic treatment and obesity. Gut Microbes.

[90] Bartlett J.G. (2010). *Clostridium difficile*: Progress and challenges. Ann. NY Acad. Sci..

[91] Britton R.A., Young V.B. (2012). Interaction between the intestinal microbiota and host in *Clostridium difficile* colonization resistance. Trends Microbiol..

[92] Chen X., Katchar K., Goldsmith J.D., Nanthakumar N., Cheknis A., Gerding D.N., Kelly C.P. (2008). A mouse model of *Clostridium difficile*-associated disease. Gastroenterology.

[93] Reeves A.E., Theriot C.M., Bergin I.L., Huffnagle G.B., Schloss P.D., Young V.B. (2011). The interplay between microbiome dynamics and pathogen dynamics in a murine model of *Clostridium difficile* Infection. Gut Microbes.

[94] Theriot C.M., Koumpouras C.C., Carlson P.E., Bergin I.I., Aronoff D.M., Young V.B. (2011). Cefoperazone-treated mice as an experimental platform to assess differential virulence of *Clostridium difficile* strains. Gut Microbes.

[95] Reeves A.E., Koenigsknecht M.J., Bergin I.L., Young V.B. (2012). Suppression of *Clostridium difficile* in the gastrointestinal tracts of germfree mice inoculated with a murine isolate from the family Lachnospiraceae. Infect. Immun..

[96] Lawley T.D., Clare S., Walker A.W., Stares M.D., Connor T.R., Raisen C., Goulding D., Rad R., Schreiber F., Brandt C. (2012). Targeted restoration of the intestinal microbiota with a simple, defined bacteriotherapy resolves relapsing *Clostridium difficile* disease in mice. PLoS Pathog..

[97] Antharam V.C., Li E.C., Ishmael A., Sharma A., Mai V., Rand K.H., Wang G.P. (2013). Intestinal dysbiosis and depletion of butyrogenic bacteria in *Clostridium difficile* infection and nosocomial diarrhea. J. Clin. Microbiol..

[98] Chang J.Y., Antonopoulos D.A., Kalra A., Tonelli A., Khalife W.T., Schmidt T.M., Young V.B. (2008). Decreased diversity of the fecal microbiome in recurrent *Clostridium difficile*-associated diarrhea. J. Infect. Dis..

[99] Hopkins M.J., Macfarlane G.T. (2002). Changes in predominant bacterial populations in human faeces with age and with Clostridium difficile infection. J. Med. Microbiol..

[100] Pérez-Cobas A.E., Artacho A., Ott S.J., Moya A., Gosalbes M.J., Latorre A. (2014). Structural and functional changes in the gut microbiota associated to *Clostridium difficile* infection. Front. Microbiol..

[101] Vincent C., Stephens D.A., Loo V.G., Edens T.J., Behr M.A., Dewar K., Manges A.R. (2013). Reductions in intestinal Clostridiales precede the development of nosocomial *Clostridium difficile* infection. Microbiome.

[102] Rea M.C., Dobson A., O’Sullivan O., Crispie F., Fouhy F., Cotter P.D., Shanahan F., Kiely B., Hill C., Ross R.P. (2011). Effect of broad- and narrow-spectrum antimicrobials on *Clostridium difficile* and microbial diversity in a model of the distal colon. Proc. Natl. Acad. Sci. USA.

[103] Cohen S.H., Gerding D.N., Johnson S., Kelly C.P., Loo V.G., McDonald L.C., Pepin J., Wilcox M.H. (2010). Society for Healthcare Epidemiology of America, Infectious Diseases Society of America. Clinical practice guidelines for *Clostridium difficile* infection in adults: 2010 Update by the society for healthcare epidemiology of America (SHEA) and the infectious diseases society of America (IDSA). Infect. Control Hosp. Epidemiol..

[104] Surawicz C.M., Brandt L.J., Binion D.G., Ananthakrishnan A.N., Curry S.R., Gilligan P.H., McFarland L.V., Mellow M., Zuckerbraun B.S. (2013). Guidelines for diagnosis, treatment, and prevention of Clostridium difficile infections. Am. J. Gastroenterol..

[105] Taur Y., Pamer E.G. (2014). Harnessing microbiota to kill a pathogen: Fixing the microbiota to treat *Clostridium difficile* infections. Nat. Med..

[106] Kelly C.P., LaMont J.T. (2008). *Clostridium difficile*—More difficult than ever. N. Engl. J. Med..

[107] Hookman P., Barkin J.S. (2009). *Clostridium difficile* associated infection, diarrhea and colitis. World J. Gastroenterol..

[108] Macfarlane S. (2014). Antibiotic treatments and microbes in the gut. Environ. Microbiol..

[109] Jank T., Ziegler M.O.P., Schulz G.E., Aktories K. (2008). Inhibition of the glucosyltransferase activity of clostridial Rho/Ras-glucosylating toxins by castanospermine. FEBS Lett..

[110] McPherson S., Rees C.J., Ellis R., Soo S., Panter S.J. (2006). Intravenous immunoglobulin for the treatment of severe, refractory, and recurrent *Clostridium difficile* diarrhea. Dis. Colon Rectum..

[111] Peterfreund G.L., Vandivier L.E., Sinha R., Marozsan A.J., Olson W.C., Zhu J., Bushman F.D. (2012). Succession in the gut microbiome following antibiotic and antibody therapies for *Clostridium difficile*. PLoS ONE.

[112] Sougioultzis S., Kyne L., Drudy D., Keates S., Maroo S., Pothoulakis C., Giannasca P.J., Lee C.K., Warny M., Monath T.P. (2005). *Clostridium difficile* toxoid vaccine in recurrent *C. difficile*-associated diarrhea. Gastroenterology.

[113] Taylor C.P., Tummala S., Molrine D., Davidson L., Farrell R.J., Lembo A., Hibberd P.L., Lowy I., Kelly C.P. (2008). Open-label, dose escalation phase I study in healthy volunteers to evaluate the safety and pharmacokinetics of a human monoclonal antibody to *Clostridium difficile* toxin A. Vaccine.

[114] Borody T.J., Warren E.F., Leis S.M., Surace R., Ashman O., Siarakas S. (2004). Bacteriotherapy using fecal flora: Toying with human motions. J. Clin. Gastroenterol..

[115] Petrof E.O., Gloor G.B., Vanner S.J., Weese S.J., Carter D., Daigneault M.C., Brown E.M., Schroeter K., Allen-Vercoe E. (2013). Stool substitute transplant therapy for the eradication of *Clostridium difficile* infection: “RePOOPulating” the gut. Microbiome.

[116] Van Nood E., Vrieze A., Nieuwdorp M., Fuentes S., Zoetendal E.G., de Vos W.M., Visser C.E., Kuijper E.J., Bartelsman J.F., Tijssen J.G. (2013). Duodenal infusion of donor feces for recurrent *Clostridium difficile*. N. Engl. J. Med..

[117] Bakken J.S., Borody T., Brandt L.J., Brill J., Demarco D.C., Franzos M.A., Kelly C., Khoruts A., Louie T., Martinelli L.P. (2011). Treating *Clostridium difficile* infection with fecal microbiota transplantation. Clin. Gastroenterol. Hepatol..

[118] Gough E., Shaikh H., Manges A.R. (2011). Systematic review of intestinal microbiota transplantation (fecal bacteriotherapy) for recurrent *Clostridium difficile* infection. Clin. Infect. Dis..

[119] Khoruts A., Dicksved J., Jansson J.K., Sadowsky M.J. (2010). Changes in the composition of the human fecal microbiome after bacteriotherapy for recurrent *Clostridium difficile*-associated diarrhea. J. Clin. Gastroenterol..

[120] Borody T.J., Khoruts A. (2012). Fecal microbiota transplantation and emerging applications. Nat. Rev. Gastroenterol. Hepatol..

[121] Kassam Z., Lee C.H., Yuan Y., Hunt R.H. (2013). Fecal microbiota transplantation for *Clostridium difficile* infection: Systematic review and meta-analysis. Am. J. Gastroenterol..

[122] Ley R.E. (2014). Harnessing microbiota to kill a pathogen: The sweet tooth of *Clostridium difficile*. Nat. Med..

[123] Kelly C.R., Ihunnah C., Fischer M., Khoruts A., Surawicz C., Afzali A., Aroniadis O., Barto A., Borody T., Giovanelli A. (2014). Fecal microbiota transplant for treatment of *Clostridium difficile* infection in immunocompromised patients. Am. J. Gastroenterol..

[124] Smith C.A., O’Maille G., Want E.J., Qin C., Trauger S.A., Brandon T.R., Custodio D.E., Abagyan R., Siuzdak G. (2005). METLIN: A metabolite mass spectral database. Ther. Drug Monit..

[125] Gerding D.N., Meyer T., Lee C., Cohen S.H., Murthy U.K., Poirier A., van Schooneveld T.C., Pardi D.S., Ramos A., Barron M.A. (2015). Administration of spores of nontoxigenic *Clostridium difficile* strain M3 for prevention of recurrent *C. difficile* infection: A randomized clinical trial. JAMA.

[126] Su W.J., Waechter M.J., Bourlioux P., Dolegeal M., Fourniat J., Mahuzier G. (1987). Role of volatile fatty acids in colonization resistance to *Clostridium difficile* in gnotobiotic mice. Infect. Immun..

[127] Sorg J.A., Sonenshein A.L. (2008). Bile salts and glycine as cogerminants for *Clostridium difficile* spores. J. Bacteriol..

[128] Wilson K.H. (1983). Efficiency of various bile salt preparations for stimulation of *Clostridium difficile* spore germination. J. Clin. Microbiol..

[129] Francis M.B., Allen C.A., Shrestha R., Sorg J.A. (2013). Bile acid recognition by the *Clostridium difficile* germinant receptor, CspC, is important for establishing infection. PLoS Pathog..

[130] Britton R.A., Young V.B. (2014). Role of the intestinal microbiota in resistance to colonization by *Clostridium difficile*. Gastroenterology.

[131] Ridlon J.M., Kang D.J., Hylemon P.B. (2006). Bile salt biotransformations by human intestinal bacteria. J. Lipid Res..

[132] Howerton A., Patra M., Abel-Santos E. (2013). A new strategy for the prevention of *Clostridium difficile* infection. J. Infect. Dis..

[133] Sorg J.A., Sonenshein A.L. (2010). Inhibiting the initiation of *Clostridium difficile* spore germination using analogs of chenodeoxycholic acid, a bile acid. J. Bacteriol..

[134] Giel J.L., Sorg J.A., Sonenshein A.L., Zhu J. (2010). Metabolism of bile salts in mice influences spore germination in *Clostridium difficile*. PLoS ONE.

[135] Theriot C.M., Koenigsknecht M.J., Carlson P.E., Hatton G.E., Nelson A.M., Li B., Huffnagle G.B., Li J.Z., Young V.B. (2014). Antibiotic-induced shifts in the mouse gut microbiome and metabolome increase susceptibility to *Clostridium difficile* infection. Nat. Commun..

[136] Begley M., Hill C., Gahan C.G.M. (2006). Bile salt hydrolase activity in probiotics. Appl. Environ. Microbiol..

[137] Ridlon J.M., Kang D.J., Hylemon P.B. (2010). Isolation and characterization of a bile acid inducible 7alpha-dehydroxylating operon in *Clostridium hylemonae* TN271. Anaerobe.

[138] Leatham M.P., Banerjee S., Autieri S.M., Mercado-Lubo R., Conway T., Cohen P.S. (2009). Precolonized human commensal *Escherichia coli* strains serve as a barrier to *E. coli* O157:H7 growth in the streptomycin-treated mouse intestine. Infect. Immun..

[139] Maltby R., Leatham-Jensen M.P., Gibson T., Cohen P.S., Conway T. (2013). Nutritional basis for colonization resistance by human commensal *Escherichia coli* strains HS and Nissle 1917 against *E. coli* O157:H7 in the mouse intestine. PLoS ONE.

[140] Wilson K.H., Perini F. (1988). Role of competition for nutrients in suppression of *Clostridium difficile* by the colonic microflora. Infect. Immun..

[141] Merrigan M., Sambol S., Johnson S., Gerding D.N. (2003). Susceptibility of hamsters to human pathogenic *Clostridium difficile* strain B1 following clindamycin, ampicillin or ceftriaxone administration. Anaerobe.

[142] Ng K.M., Ferreyra J.A., Higginbottom S.K., Lynch J.B., Kashyap P.C., Gopinath S., Naidu N., Choudhury B., Weimer B.C., Monack D.M. (2013). Microbiota-liberated host sugars facilitate post-antibiotic expansion of enteric pathogens. Nature.

[143] Sambol S.P., Merrigan M.M., Tang J.K., Johnson S., Gerding D.N. (2002). Colonization for the prevention of *Clostridium difficile* disease in hamsters. J. Infect. Dis..

[144] Rea M.C., Sit C.S., Clayton E., O’Connor P.M., Whittal R.M., Zheng J., Vederas J.C., Ross R.P., Hill C. (2010). Thuricin CD, a posttranslationally modified bacteriocin with a narrow spectrum of activity against *Clostridium difficile*. Proc. Natl. Acad. Sci. USA.

[145] Hasegawa M., Kamada N., Jiao Y., Liu M.Z., Núñez G., Inohara N. (2012). Protective role of commensals against *Clostridium difficile* infection via an IL-1β-mediated positive-feedback loop. J. Immunol..

[146] Hasegawa M., Yamazaki T., Kamada N., Tawaratsumida K., Kim Y.G., Núñez G., Inohara N. (2011). Nucleotide-binding oligomerization domain 1 mediates recognition of *Clostridium difficile* and induces neutrophil recruitment and protection against the pathogen. J. Immunol..

[147] Jarchum I., Liu M., Shi C., Equinda M., Pamer E.G. (2012). Critical role for MyD88-mediated neutrophil recruitment during *Clostridium difficile* colitis. Infect. Immun..

[148] Brandl K., Plitas G., Mihu C.N., Ubeda C., Jia T., Fleisher M., Schnabl B., DeMatteo R.P., Pamer E.G. (2008). Vancomycin-resistant enterococci exploit antibiotic-induced innate immune deficits. Nature.

[149] Atarashi K., Tanoue T., Shima T., Imaoka A., Kuwahara T., Momose Y., Cheng G., Yamasaki S., Saito T., Ohba Y. (2011). Induction of colonic regulatory T cells by indigenous *Clostridium* species. Science.

[150] Bibbò S., Lopetuso L.R., Ianiro G., di Rienzo T., Gasbarrini A., Cammarota G. (2014). Role of microbiota and innate immunity in recurrent *Clostridium difficile* infection. J. Immunol. Res..

[151] Lopetuso L.R., Scaldaferri F., Petito V., Gasbarrini A. (2013). Commensal Clostridia: Leading players in the maintenance of gut homeostasis. Gut Pathog..

[152] Umesaki Y., Setoyama H., Matsumoto S., Imaoka A., Itoh K. (1999). Differential roles of segmented filamentous bacteria and clostridia in development of the intestinal immune system. Infect. Immun..

[153] Dai Z.L., Wu G., Zhu W.Y. (2011). Amino acid metabolism in intestinal bacteria: Links between gut ecology and host health. Front. Biosci..

[154] Kibe R., Kurihara S., Sakai Y., Suzuki H., Ooga T., Sawaki E., Muramatsu K., Nakamura A., Yamashita A., Kitada Y. (2014). Upregulation of colonic luminal polyamines produced by intestinal microbiota delays senescence in mice. Sci. Rep..

[155] Rojo D., Gosalbes M.J., Ferrari R., Pérez-Cobas A.E., Hernández E., Oltra R., Buesa J., Latorre A., Barbas C., Ferrer M. (2015). ISME J..

